# New Trends in Pathology: From Cell Morphology to Molecular Medicine

**DOI:** 10.3390/ijms241411743

**Published:** 2023-07-21

**Authors:** Maria Addolorata Bonifacio, Maria Addolorata Mariggiò

**Affiliations:** Department of Precision and Regenerative Medicine and Ionian Area, University of Bari Aldo Moro Medical School, 70124 Bari, Italy; m.bonifacio@studenti.uniba.it

## 1. A Commemorative Issue in Honour of Rudolf Virchow

After Rudolf Virchow’s pioneering works, technological advances boosted the scientific interest in this research field, which nowadays is still far from extinguished. A quick search on Scopus shows more than 100,000 papers with the keywords “molecular” and “pathology”. Of these papers, 6163 were published only in 2022. Its strong translational calling makes molecular pathology a hot topic of current scientific literature, with its ultimate goal of improving diagnostic tools. In this respect, one of the greatest achievements in this research field is represented by nucleic acid-based techniques in oncohematology, which resulted in more precise diagnoses and, thus, in personalised therapies. Nevertheless, molecular pathology is not only sequencing and PCR. Indeed, combined spectrometric and chromatographic methods are reshaping the detection of disease markers, opening new landscapes in complex disease diagnosis and follow-up of genetic disorders, metabolic alterations and infectious diseases. Meanwhile, diagnostic techniques are becoming increasingly closer to the patient’s bedside, being implemented in routine analyses facilities. In addition, powerful diagnostic tools are being marketed in easy-to-use, cartridge-based tests, allowing small clinical centres to handle complex clinical conditions.

**Figure 1:**
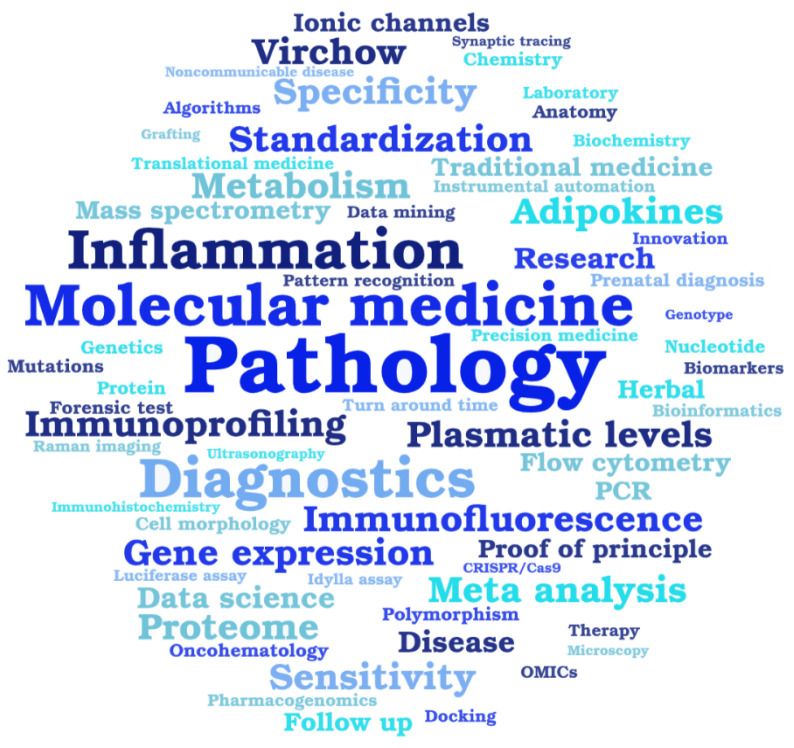
A word cloud relevant to the papers published in this Special Issue.

This Special Issue aimed to honour the memory of Rudolf Virchow, collecting updated reviews and original papers dealing with the most advanced concepts in molecular pathology. The Special Issue collected 32 papers (22 original research articles and 10 reviews), with already more than 30 overall citations, demonstrating the strong interest of the scientific community for the selected topic. Therefore, a second edition of the same Special Issue has already been launched, aiming at further collecting other contributions relevant to molecular pathology research, helping academics, clinicians and laboratory experts to stay tuned with the latest news in this buzzing research field (Figure 1).

The current urgent need of standardised diagnostic criteria, as well as of timely and effective biomarkers, was highlighted by several papers published in this Special Issue. In particular, Eijssen and coworkers reviewed the pathophysiology, treatment options and available biomarkers relevant to post-traumatic stress disease [1]. Indeed, neuropsychiatric conditions and noncommunicable diseases strongly require reliable biomarkers to predict susceptibility, aid diagnosis and monitor treatment, as also encouraged by the latest EU4Health actions.

Similarly, the research of reliable biomarkers was highlighted in the study of Szewczyk-Golec and colleagues, who shed light on head and neck carcinogenesis [2]. The authors tested 46 patients with head and neck cancer, observing an impaired glucose metabolism and the disruption of adipose tissue’s endocrine function during carcinogenesis. In addition, looking for effective biomarkers, the authors studied serum levels of particular adipokines (i.e., glucagon, plasminogen activator inhibitor-1, visfatin, adipsin and ghrelin), finding a potential correlation with the pathogenesis of head and neck cancer. Furthermore, obesity seemed to affect metabolic impairment linked to carcinogenesis.

The impact of obesity was also the focus of the paper published by De Hert et al., who demonstrated a correlation between the expression of prolyl carboxypeptidase (PRCP) and obesity in men affected by type 2 diabetes [3]. Moreover, beyond PRCP serum levels, the authors observed, for the first time, PRCP expression in human adipose tissue, specifically in the visceral district, rather than in the subcutaneous adipose tissue. They hypothesised that immune cells residing in visceral adipose tissue may be responsible for the high PRCP serum levels detected in diabetic, obese men.

On the other hand, Rossi et al. aimed at improving the diagnostic screening and therapy monitoring of Duchenne muscular dystrophy (DMD), identifying a biomarker more specific than creatine kinase [4]. Therefore, by exploiting a suspension bead immunoassay, the authors detected dystrophin protein fragments in a small cohort of human plasma samples, proposing a proof-of-concept to be further confirmed on a larger set of samples.

Through dystrophin protein fragments, the authors were able to distinguish DMD patients from healthy individuals and from people affected by other neuromuscular diseases. The obtained results were validated with targeted liquid chromatography mass spectrometry.

Meanwhile, Gentile and coworkers performed a systematic review on sickle cell disease, investigating the significance of homocysteine plasmatic levels and TT genotype relevant to methylenetetrahydrofolate (MTHFR) [5]. Their meta-analysis emphasised the presence of higher levels of plasma homocysteine in children with sickle cell disease from India and Middle East, likely because of a vitamin-poor diet. Conversely, the occurrence of MTHFR TT genotype was linked to ischemic stroke complications in adults affected by the same genetic condition.

Heart diseases were the focus of the manuscript by Andriulé et al., who studied the expression of transient receptor potential melastatin ion channels (TRPM) in heart tissues of healthy pigs and humans [6]. In addition, the authors examined cardiac tissues belonging to human ischemic hearts and atrial fibrillation hearts, finding immunofluorescence signals indicating an upregulation of TRPM6 and TRPM7 in diseased samples. The availability of TRPM modulators could represent a new avenue to treat atrial diseases.

Similarly, Reis and coworkers studied 105 patients with nonvalvular atrial fibrillation, looking for relevant inflammatory biomarkers [7]. Through cytometric bead array and multiplex immunoassay, they confirmed the correlation between atrial fibrillation and plasmatic levels of IL-6, IL-10 and TNF, while discovering a new potential inflammatory marker, i.e., interferon-gamma-induced protein (IP-10).

Chronic inflammation was also the main topic studied by Moysidou et al., who exploited flow cytometry techniques to provide interesting insights into immunosenescent profiles caused by two different chronic inflammatory diseases, i.e., systemic lupus erythematosus (SLE) and end-stage kidney disease (ESKD) [8]. A significant lymphopenia was observed in both SLE and ESKD patients on haemodialysis, compared to healthy controls. Moreover, lymphopenia occurred more often in the B-cell compartment during SLE, while it mainly interested T-lymphocytes in patients with ESKD on haemodialysis.

In a subsequent work, the same authors dissected T lymphocytes subpopulations in patients affected by another chronic inflammatory condition, i.e., lupus nephritis (LN) [9]. Comparing the immune profile of 30 patients with 20 healthy controls, Lioulios et al. showed that exhausted T lymphocyte subpopulations predominated within LN patients, while the T cell phenotype was related to the disease stage.

The opportunity to exploit Raman spectroscopy to study macrophage populations was investigated by Naumann et al., who described the use of single cell Raman imaging to distinguish between pro-inflammatory M1, anti-inflammatory M2 and resting M0 macrophage phenotypes [10]. The proposed approach had the advantages to be label-free and non-destructive. Furthermore, the accuracy of Raman spectroscopy was also validated through flow cytometry and fluorescence imaging, using monocytes from healthy donors in vitro differentiated in M0, M1 and M2 macrophage phenotypes.

Similarly, Fisseler-Eckhoff and colleagues tested the accuracy, sensitivity and specificity of the Idylla IVD assays, describing the performances of these diagnostic tools on DNA/RNA samples, as well as on archival formalin-fixed paraffin-embedded tissues (FFPE) [11]. These automated, rapid molecular analyses also achieved excellent outcomes (100% specificity and accuracy above 96%) with sample types not recommended by the manufacturer. Thus, Idylla assays represent reliable tools for qualitative and quantitative cancer diagnostics.

Another study on the accuracy, sensitivity and diagnostic odds ratio of an instrumental diagnostic technique, i.e., ultrasonography, was proposed by Issa et al. [12]. The authors reviewed the effectiveness of ultrasonography in detecting extrathyroidal extension in patients with papillary thyroid carcinoma. The meta-analysis involved a total of 3795 cancer patients, described in 11 studies. Extrathyroidal extension was detected with a specificity of 51% and a specificity of 76%, resulting adequate for diagnostic purposes. Moreover, the authors discussed the opportunity to combine other imaging analyses to ultrasonography, as well as genetic assessments, to provide clinicians with a more complete diagnostic profile, thus driving customised treatment strategies.

Aiming to overcome current treatment limitations, Adam and coworkers described an innovative preservation solution (IGL-2) to allow transplantation of marginal grafts from livers with steatosis [13]. The authors tested the combination of IGL-2 with static cold storage and hypothermic oxygenated perfusion on rats. Thus, analysing perfusates, the authors found lower levels of glycocalyx proteins, transaminases and caspase 3, demonstrating the protective features of IGL-2 solution on fatty livers.

Meanwhile, Wang et al. explored the complex odour processing mechanisms in Thy1-Cre mice, through rabies virus-mediated retrograde monosynaptic tracing [14]. With this approach, the authors were able to clarify the architecture of centrifugal connections between central brain regions and two selected types of neurons within the olfactory bulb, i.e., granule cells and mitral/tufted cells. Their findings confirmed the pivotal role played by centrifugal projections in olfactory processing.

On the other hand, Koszewicz and coworkers aimed at assessing damages occurring in different neuron regions and the subsequent chronic inflammatory demyelinating polyradiculoneuropathy (CIDP), as well as the disorders related to this condition [15]. The authors dissected the molecular differences within Ranvier nodes, juxtaparanode region and initial axon segments, reporting the detailed mechanisms responsible for CIDP outbreak.

Catching a transmissible pathologic memory code was the main goal of the hypothesis paper published by Berlanga-Acosta et al., who administered cell-free filtrates, belonging to human tissues affected by cancer, diabetes or arteriosclerosis tissues, to healthy animals [16]. The authors observed the onset of pathological features (i.e., nervous and cutaneous alterations typical of type 2 diabetes, vasal thickening peculiar of arteriosclerosis and pre-carcinogenic lesions in mesenchymal and epithelial tissues).

Atherosclerosis pathogenic mechanisms and the impact of inflammation were the main topic of the review article by Vancheri and colleagues [17]. The authors clarified the consequences of an aberrant sympathetic activation on the release of inflammatory mediators, able to boost cardiovascular adverse events such as stroke and myocardial infarction. Moreover, an interesting insight was provided on the connection of chronic psychological stress and inflammation.

Similarly, Fortingo et al. reviewed the role of acute and chronic inflammation during corneal wound healing [18]. The authors highlighted the benefits of microbe-triggered, acute inflammation in preventing infections and supporting tissue remodelling. On the other hand, they pointed out the disadvantages of a chronic, excessive inflammatory activation, which may delay wound healing processes.

In this respect, Im and colleagues presented the advantages of atractylodin, a natural anti-inflammatory molecule isolated from the oriental herbal medicine *Atractylodes lancea*, traditionally exploited to treat gastrointestinal diseases [19]. The authors focused on the molecular targets of atractylodin, combining in silico, in vitro and in vivo studies to demonstrate its ability to bind the peroxisome proliferator-activated receptor alpha (PPARα).

It was another interaction, the one between actin fibres and CIC-5 protein, the main topic of the studies carried out by Priante et al. [20]. The authors shed light on the impact of CIC-5 protein downregulation on endocytotic processes within podocytes. They found that ClC-5 loss caused cytoskeleton derangement and impaired nephrin recycling. These outcomes were essential to sustain the hypothesis that the onset of type 1 Dent disease could be due to a podocyte disfunction, in addition to the glomerular damage subsequent to the tubulopathy.

Conversely, the molecular mechanisms underlying ethanol promotion of oral and oropharyngeal squamous cell carcinoma were studied by Shin and coworkers [21]. They clarified that nuclear factor of activated T-cells (NFAT) signalling pathway played the main role in supporting cancer stem cells proliferation, migration and metabolic activity. The latter, after chronic exposure to ethanol, was characterised by an increase in aerobic glycolysis and aldehyde dehydrogenase activity, suggesting a tight link between metabolic alterations and cancer stemness.

Meanwhile, Wilson et al. investigated the overall incidence of cancer, as well as its mortality-associated rate, in patients who received bariatric surgery or conventional treatment [22]. The meta-analysis involved 32 studies and was performed through the inverse variance method and random effects model. The long-term weight loss subsequent to bariatric surgery seemed to discourage carcinogenesis, ameliorating patients’ metabolism. Concerning specific cancers, after bariatric surgery, the incidences of colorectal, breast, pancreatic, ovarian, gallbladder and endometrial cancer, as well as of hepatocellular carcinoma, were significantly reduced. A positive impact of the surgical treatment on metabolic syndrome was suggested by the authors.

In addition, Čičin-Šain and colleagues [23] explored the metabolic response of high and low serotonin tones in rats exposed to an obesogenic diet. Rat sublines with constitutionally low serotonin levels were more subjected to major metabolic alterations, insulin resistance and body weight gain. On the other hand, the high serotonin rat subline seemed to be protected from adverse metabolic outcomes, providing useful clues to control metabolic disorders in obese people.

Another powerful molecule able to regulate fat metabolism is leptin, an adipokine mainly synthesised by adipose tissue. Casado et al. reviewed the impact of leptin on the homeostasis of bone density and muscle mass [24]. Furthermore, the authors discussed central leptin resistance in neurological conditions, i.e., Parkinson’s and Alzheimer’s diseases.

A potential strategy for colorectal cancer prevention and treatment was the topic studied by Zakaria and coworkers [25]. The authors analysed by UHPLC-QTOF-MS the anticancer, anti-obesity and anti-adipogenic features of the ethanolic extract of *Andrographis paniculata*, an oriental plant used in traditional medicine. Colorectal cancer was induced in Sprague Dawley rats administering 1,2-dimethylhydrazine. Then, the rats were exposed to a high-fat diet and to the plant extract. The latter significantly reduced the number of aberrant crypt loci and ameliorated colonic dysplasia.

An epithelial to mesenchymal transition was presented by Droździk et al. in gingival overgrowth subsequent to administration of drugs altering intracellular calcium transport (i.e., phenytoin, nifedipine, cyclosporin) [26]. The authors reviewed the molecular mechanisms underlying drug-induced gingival overgrowth (DIGO), pointing out the role of some drugs in triggering the massive synthesis of collagen and glycosaminoglycans, characterizing the extracellular matrix of connective tissues. At the same time, DIGO was due to an impaired tissue regeneration and remodelling, subsequent to the disruption of integrin-mediated interactions and collagenase activity.

On the other hand, Pozzolini and coworkers observed an enhancement in regenerative processes of the sponge *Chondrosia reniformis* after exposure to a specific near-infrared light [27]. Exploring gene expression profiles of controls, as well as of *C. reniformis* specimens exposed to light, the authors clarified the upregulation of TGF3 and TGF6 in the early regeneration stages, in addition to the delayed overexpression of TGF5. These outcomes support the hypothesis that advantageous light interactions are evolutionarily conserved within several, different organisms.

The opportunity to treat pregnant women with pre-eclampsia with aspirin was investigated by Walsh et al. [28]. Indeed, the authors highlighted the responsiveness of pre-eclamptic neutrophils to protease activation. Thus, tracing the molecular pathway of nuclear factor-kappa beta (NF-κβ), this work displayed its return in the cytoplasmic compartment and the subsequent decrease in thromboxane synthesis, due to the downstream inhibition of cyclooxygenase-2. Therefore, aspirin could be an interesting therapeutic option to treat protease-mediated inflammation in pre-eclamptic women.

While aspirin could hinder adverse events triggered by cell-mediated immune response, Pugliatti and colleagues reported the potential of dimethylfumarate (DMF) to decrease humoral immune response in multiple sclerosis patients [29]. Their paper described a pilot study performed on 17 patients, in which DMF reduced the levels of antibodies directed against the capsid antigen of Epstein–Barr Virus, associated with multiple sclerosis onset.

Similarly, Zhou et al. described the grafting of a modified, single domain antibody against SARS-CoV-2 on a liposome surface [30]. An effective neutralization activity was observed in vitro. Therefore, the authors exploited the modified liposome, also embedding cGAMP, as a preventive vaccine against viral infection. Successful immunization was reported in BALB/c male mice.

The fight against SARS-CoV-2 was also the main focus of the work published by Nishida and coworkers [31]. The authors suggested a strategy to hinder viral damage on cardiomyocytes, based on the inhibition of the protein complex TRPC3-Nox2 (transient receptor potential canonical 3 and NADPH oxidase 2). Indeed, this complex enhances viral entry through angiotensin-converting enzyme receptor (ACE 2). Ibudilast was able to inhibit TRPC3-Nox2 complex formation, triggered by ATP release from pannexin channels, avoiding cardiomyocytes metabolic and contraction alterations.

Conversely, Huang et al. explored the opportunity to treat pore-forming, toxin-induced diseases with statins [32]. In particular, the cytotoxic action of epsilon toxin produced by *Clostridium perfringens* was assessed in vitro in the presence of zaragozic acid. The latter statin was able to reduce the amount of lipid rafts on the membranes of Madin–Darby canine kidney cells, hindering pores formation by epsilon toxin.

## 2. Open Challenges and Future Perspectives

Each paper belonging to this Special Issue highlighted a common take-home-message, i.e., the need of an integrated work strategy to achieve true innovation. Indeed, a multidisciplinary approach is required to develop cutting-edge diagnostic techniques. In this respect, it is compulsory to think “outside the box”, through collaboration among scientists with complementary skills. The young pathology researchers should bear in mind a translational imperative, i.e., embrace complexity, engineer versatility, deliver simplicity. The continuous, strong link between the application-oriented clinical perspective and the academic background of scientists still constitutes the main path of molecular pathology research, whatever challenges the future holds. According to Virchow’s insights, we strongly believe that the reported advancements in molecular pathology will open up new landscapes in diagnosis, treatment and monitoring of diseases.
